# Endogenous and Exogenous Estrogen Exposures: How Women’s Reproductive Health Can Drive Brain Aging and Inform Alzheimer’s Prevention

**DOI:** 10.3389/fnagi.2022.831807

**Published:** 2022-03-09

**Authors:** Steven Jett, Niharika Malviya, Eva Schelbaum, Grace Jang, Eva Jahan, Katherine Clancy, Hollie Hristov, Silky Pahlajani, Kellyann Niotis, Susan Loeb-Zeitlin, Yelena Havryliuk, Richard Isaacson, Roberta Diaz Brinton, Lisa Mosconi

**Affiliations:** ^1^Department of Neurology, Weill Cornell Medical College, New York, NY, United States; ^2^Department of Radiology, Weill Cornell Medical College, New York, NY, United States; ^3^Department of Obstetrics and Gynecology, Weill Cornell Medical College, New York, NY, United States; ^4^Department of Pharmacology, University of Arizona, Tucson, AZ, United States; ^5^Department of Neurology, University of Arizona, Tucson, AZ, United States

**Keywords:** hormones, sex differences, menopause, reproductive history, risk factors, Alzheimer’s disease

## Abstract

After advanced age, female sex is the major risk factor for late-onset Alzheimer’s disease (AD), the most common cause of dementia affecting over 24 million people worldwide. The prevalence of AD is higher in women than in men, with postmenopausal women accounting for over 60% of all those affected. While most research has focused on gender-combined risk, emerging data indicate sex and gender differences in AD pathophysiology, onset, and progression, which may help account for the higher prevalence in women. Notably, AD-related brain changes develop during a 10–20 year prodromal phase originating in midlife, thus proximate with the hormonal transitions of endocrine aging characteristic of the menopause transition in women. Preclinical evidence for neuroprotective effects of gonadal sex steroid hormones, especially 17β-estradiol, strongly argue for associations between female fertility, reproductive history, and AD risk. The level of gonadal hormones to which the female brain is exposed changes considerably across the lifespan, with relevance to AD risk. However, the neurobiological consequences of hormonal fluctuations, as well as that of hormone therapies, are yet to be fully understood. Epidemiological studies have yielded contrasting results of protective, deleterious and null effects of estrogen exposure on dementia risk. In contrast, brain imaging studies provide encouraging evidence for positive associations between greater cumulative lifetime estrogen exposure and lower AD risk in women, whereas estrogen deprivation is associated with negative consequences on brain structure, function, and biochemistry. Herein, we review the existing literature and evaluate the strength of observed associations between female-specific reproductive health factors and AD risk in women, with a focus on the role of endogenous and exogenous estrogen exposures as a key underlying mechanism. Chief among these variables are reproductive lifespan, menopause status, type of menopause (spontaneous vs. induced), number of pregnancies, and exposure to hormonal therapy, including hormonal contraceptives, hormonal therapy for menopause, and anti-estrogen treatment. As aging is the greatest risk factor for AD followed by female sex, understanding sex-specific biological pathways through which reproductive history modulates brain aging is crucial to inform preventative and therapeutic strategies for AD.

## Introduction

### Alzheimer’s Disease and the Importance of Being Female

Alzheimer’s disease (AD) is the most common cause of dementia and the sixth leading cause of death in Western societies, affecting over 24 million patients worldwide (Alzheimer’s Association, 2021). Today, AD remains the only major cause of mortality without an effective disease-modifying treatment. Given lack of therapeutics to prevent, delay or reverse late-onset AD, the number of persons living with AD dementia is projected to nearly triple by 2050 (Alzheimer’s Association, 2021), placing a considerable burden on public health systems. The limited success of disease-modifying trials is likely due to testing of potential therapeutic agents too late in disease course, and to an incomplete understanding of the complex pathophysiological mechanisms underlying AD (Andrieu et al., 2015).

These shortcomings are attributable, at least in part, to the fact that most research has ignored the existence of biological sex differences in AD and focused on sex-aggregated risk. Given increasing evidence that AD prevalence, symptomatology, and risk profiles vary by sex (Mielke et al., 2014; Snyder et al., 2016; Ferretti et al., 2018; Rahman et al., 2019), a possible strategy to stem the AD epidemic is earlier intervention coupled with sex-specific interventions.

Female sex is the second most significant risk factor for AD after advanced age (Farrer et al., 1997). AD affects more women than men, with a nearly 2:1 ratio in many countries (Alzheimer’s Association, 2021), and with postmenopausal women accounting for over 60% of all those affected (Brookmeyer et al., 1998). This disparity may be a consequence of women’s relatively longer life expectancy (Nebel et al., 2018), coupled with selective survival of men with higher cardiovascular health (Chêne et al., 2015). However, evidence against this hypothesis stems from multiple sources indicating a greater age-adjusted risk of AD in women independent of survival rates (Gao et al., 1998; Andersen et al., 1999; Lobo et al., 2000; Carter et al., 2012), as well as faster rates of conversion from mild cognitive impairment (MCI) to AD irrespective of age and educational level (Holland et al., 2013; Lin and Doraiswamy, 2014; Tifratene et al., 2015; Gamberger et al., 2017). Women are also more likely to develop amnestic MCI, often a prodromal stage to AD dementia (Petersen, 2004), while men exhibit a higher incidence of non-amnestic MCI, which is more closely associated with non-AD dementias (Caracciolo et al., 2008; Roberts et al., 2012).

Sex differences in cognitive functions affected by AD have also been documented, as men tend to have higher visuospatial and motor coordination scores than women (McCarrey et al., 2016), while women exhibit higher verbal memory scores than men even after a diagnosis of early AD (Sundermann et al., 2016a,b; Rentz et al., 2017). There is concern that the female advantage on memory testing might mask AD-related brain changes, resulting in women being diagnosed at a later stage than their male counterparts. Support to this hypothesis comes from AD biomarker studies, including biomarkers of AD pathology, such as Aβ accumulation on PET imaging and in CSF; biomarkers of neurodegeneration, including biomarkers of neuronal injury and degeneration such as increased CSF tau levels and structural MRI measures of cerebral atrophy and gray matter (GM) volume; and biomarkers of synaptic dysfunction, such as lower cerebral glucose metabolism (CMRglc) on 2-deoxy-2-[^18^F]fluoro-d-glucose positron emission tomography (FDG PET) (Jack et al., 2013). Generally, Aβ biomarkers are regarded as providing the highest specificity for AD, whereas biomarkers of neurodegeneration are not specific to AD but show higher sensitivity to AD-related changes, while also correlating with cognitive declines (Jack et al., 2013). Where Aβ biomarkers are specific to AD, CMRglc and GM volume are indirect measurements of synaptic and neuronal loss (Bobinski et al., 2000). Nonetheless, combined imaging and autopsy studies show good correlation between ante-mortem FDG-PET diagnosis and post-mortem confirmation of AD (Hoffman et al., 2000), and between the degree of GM volume loss and Braak staging (Jack et al., 2002; Silbert et al., 2003). Additionally, reductions in CMRglc and GM volume were shown to precede and correlate with declines in cognitive performance along the continuum from normal cognition to MCI to AD dementia, and predict conversion to dementia with high accuracy (de Leon et al., 2001; Drzezga et al., 2003; Mosconi et al., 2004, 2008, 2009; Jack et al., 2013). Biomarker studies, especially by means of brain imaging techniques, indicate that female AD patients exhibit greater rates of neurodegenerative decline, as evidenced by increased hippocampal atrophy and greater neurofibrillary tangle burden, relative to male AD patients with the same level of brain amyloid-β (Aβ) load, with no difference in lifetime AD risk (Barnes et al., 2005; Buckley et al., 2018). Non-demented elderly women with biomarker-determined Aβ positivity also show more rapid hippocampal volume loss than men with similar pathological burden (Hua et al., 2010; Skup et al., 2011; Ardekani et al., 2016; Koran et al., 2017). As reviewed below, cognitively normal midlife women at risk for AD (e.g., family history of late-onset AD and/or heterozygous or homozygous for APOE-4 allele) exhibit increased indicators of AD risk as compared to age-controlled men, including higher Aβ load, lower CMRglc, and lower GM and white matter (WM) volume (Mosconi et al., 2017a,b, 2018, 2021; Rahman et al., 2020).

Sex differences in the effects of apolipoprotein E (APOE) epsilon 4 allele, the strongest genetic risk factor for late-onset AD, have also been documented. The APOE-4 allele has been associated with a younger age at AD onset, and increased Aβ deposition, with greatest risk observed in those carrying two alleles (for recent review, see Yamazaki et al., 2019; Husain et al., 2021). Female APOE-4 carriers are more likely than male carriers to develop AD, with a nearly 4- and 10-fold in women with one and two APOE-4 alleles, respectively, whereas men exhibit essentially little increased risk with one APOE-4 allele and a fourfold increased risk with two APOE-4 alleles (Farrer et al., 1997; Altmann et al., 2014; Ungar et al., 2014). Women carrying the APOE-4 genotype also exhibit higher Aβ deposition (Mosconi et al., 2017b), reduced brain connectivity (Fleisher et al., 2005; Damoiseaux et al., 2012; Mosconi et al., 2017b), greater brain hypometabolism, hippocampal atrophy and cortical thinning (Fleisher et al., 2005; Altmann et al., 2014; Sampedro et al., 2015), and greater levels of tau protein in CSF (Altmann et al., 2014; Hohman et al., 2018) as compared to genotype-controlled men.

Overall, while age and lifespan certainly play a role, there is increasing recognition that female sex, along with additional genetic, social, and lifestyle factors, is an important risk factor for AD (Mielke et al., 2014; Ferretti et al., 2018; Rahman et al., 2019). These observations have spurred renewed interest in investigating sex differences in the biological mechanisms underlying AD. Identifying endophenotypes of AD risk that emerge early in the course of the disease and that differ by sex is vital to clarify the mechanistic pathways linking female-specific physiological processes to AD, and to identify sex-specific targets for risk reduction and therapeutic development.

### From Bedside to Bench

The pathophysiological process of AD, including accumulation of Aβ plaques, neurofibrillary tangles, neuronal and synaptic loss, begins 10–20 years prior to clinically detectable symptoms (Sperling et al., 2011). Therefore, the prodromal period starts as early as midlife, which coincides with the hormonal transitions of endocrine aging characteristic of the menopause transition in women (Brinton et al., 2015).

The menopause transition is a complex neuro-endocrine transitional state marked by changes in gonadal sex steroid hormones, especially 17β-estradiol, following progressive oocyte depletion. While menopause is primarily associated with reproductive senescence, it has wide ranging neurological consequences including changes in thermoregulation manifesting as hot flashes, circadian rhythm disruption manifesting as sleep disturbances, mood changes, as well as memory and attention complaints (Brinton et al., 2015). Many of these symptoms are risk factors for AD in turn (Livingston et al., 2020). The emergence of multiple AD risks in midlife, both at the neurophysiological level and at the clinical symptomatic level, are consistent with menopause being a tipping point for AD risk in later life (Brinton et al., 2015; Scheyer et al., 2018; Rahman et al., 2019).

A burgeoning array of preclinical studies has provided evidence for neuroprotective effects of estrogens, and identified life-time estrogen exposure as a modulator of cognitive aging in females (Morrison et al., 2006; Brinton, 2008; Brinton et al., 2015). Translational neuroimaging studies of midlife women revealed emergence of AD endophenotypes, including greater Aβ burden, glucose hypometabolism, and gray (GM) and white matter (WM) volume loss, during the menopause transition (Mosconi et al., 2017a,b, 2018, 2021; Rahman et al., 2020). Women undergoing surgically induced menopause due to salpingo-oophorectomies also exhibit increased neuropathology and imaging biomarker indicators of AD (Bove et al., 2014) at an even younger age (Zeydan et al., 2019). This suggests that estrogen deprivation following menopause may trigger or exacerbate a pre-existing disposition for AD (Morrison et al., 2006; Brinton, 2008; Brinton et al., 2015; Rahman et al., 2019).

Even before menopause, the level of gonadal hormones to which the female brain is exposed changes considerably across the lifespan. Estrogen levels in women can fluctuate widely during reproductive events, and in response to both endogenous and exogenous estrogen exposures (Davis et al., 2015). Endogenous levels of estrogen vary based on several factors, including menopause status, age at menarche, age at menopause, the reproductive window, number of pregnancies and children, and gynecological surgeries. Exogenous levels of estrogen vary chiefly due to use of hormonal contraceptives (HC), menopause hormonal therapy (HT), and anti-estrogen therapies for neoplastic conditions such as ovarian and breast cancers. Gender-affirming therapy can also include hormone treatment (Winter et al., 2016).

Brain modifications resulting from reproductive health history events as related to estrogen exposure and their implications for cognitive aging and AD risk are the focus of this review. Herein, we evaluate the existing literature and assess the strength of observed associations between reproductive history factors and AD, with a focus on the role of estrogen exposure as an underlying mechanism. We conducted a literature search on the PubMed Medline and Web of Science databases for papers (excluding case-reports) published between the years 1985 and 2021, in English language, using search terms for exposures [“estrogen”, “hormones”, “menopause”, “perimenopause”, “menopause transition”, “reproductive history”, “reproductive span”, “menarche”, “parity”, “children”, “pregnancies”] and for outcomes [“Alzheimer’s disease”, “dementia”, “cognition”, “cognitive performance”] in the title or abstract. We also provided a general overview of hormonal treatments’ effects on AD risk. Knowledge of reproductive history effects on AD risk is critical to informing clinicians on management of modifiable factors for cognitive decline and for development of therapeutic targets that modify estrogenic risk factors.

## Estrogen Function in Brain

Besides their obvious role in reproduction, sex hormones are known regulators of neuronal morphology, number, and function, which makes endocrine aging an important contributor to brain aging (McEwen, 1981; Behl, 2002; Arevalo et al., 2015). The transition to menopause is marked by radical changes in the production and activity of sex steroid hormones in the body and brain (McEwen et al., 1997). As such, changes in gonadal hormones, primarily 17β-estradiol, the most potent form of estrogen and the primary circulating hormone during a woman’s reproductive years, have been proposed as major contributors to the higher risk of AD in women (Morrison et al., 2006; Brinton, 2008; Brinton et al., 2015; Rahman et al., 2019).

Estrogen is synthesized primarily in the ovaries, where it regulates the menstrual cycle. However, it is also synthesized in several non-reproductive tissues, including brain, liver, and adipose fat (Cui et al., 2013). While peripheral estrogen can cross the blood brain barrier and act on central estrogen receptors located on neurons and glia, much of the estrogen present in brain is synthesized locally (Cui et al., 2013). During development, adult life, and aging, estradiol exerts multiple regulatory actions in the central nervous system (CNS), which are mediated by direct effects on neurons and glial cells. Estrogenic input on neurons influences higher cognitive function, pain, fine motor skills, mood, susceptibility to seizures, and neuroprotection in response to brain damage (McEwen et al., 2001; Behl, 2002; McEwen, 2002; Cui et al., 2013; Arevalo et al., 2015). On glial cells, including oligodendroglia, astroglia, and microglia, estrogen is crucial for regulation of neuronal metabolism and activity as well as synaptic transmission and plasticity (Micevych et al., 2010).

Estrogen signaling in the brain activates multiple functions through a network of receptors expressed in select cellular populations (Li et al., 2014). There are two broad classes of estrogen receptors: classical nuclear receptors, composed of alpha and beta subtypes (ERα and ERβ), and G-protein coupled estrogen receptors (GPER). ERα and ERβ are ligand-activated transcription factors that dimerize and translocate to the nucleus after ligand binding (Mannella and Brinton, 2006; Rettberg et al., 2014). Once in the nucleus, they bind to estrogen response elements (EREs) in the promoters of target genes to regulate transcription and gene expression (Björnström and Sjöberg, 2005). Membrane receptor GPERs also regulate transcription of target genes, but through indirect means via MAPK and PI3K activation and cAMP production (Li et al., 2014). The chief distinction is that ERα and ERβ lead to transcription of late response genes while GPER mediate many of the rapid responses of estradiol by means of fast signaling transcriptional activation. All types of estrogen receptors are selectively distributed throughout the brain and occur in specific nuclei and cell types. ERα and ERβ are co-expressed in the hypothalamus, amygdala, and hippocampus (Osterlund and Hurd, 2001; Hedges et al., 2012; Barth et al., 2015). However, ERα is predominant in hypothalamic nuclei that control reproduction, sexual behavior, and appetite (Osterlund and Hurd, 2001; Hedges et al., 2012; Barth et al., 2015), while ERβ is predominant in non-reproductive hypothalamic nuclei as well as in dorsal raphe nuclei, basal forebrain, prefrontal cortex, various temporal and parietal regions, posterior cingulate, and cerebellum (Mitra et al., 2003; Sugiyama et al., 2010). While ERα and ERβ both contribute to the neuroprotective effects of estrogen, ERβ plays a larger role than ERα in supporting cognition by mediating neural plasticity, regulating brain-derived neurotrophic factor (BDNF), and promoting learning and memory (Zhao et al., 2015), whereas ERα is the primary mediator of steroid induced neuroprotection, with known effects on neurovascular function and myelin repair (Dubal et al., 2001; Duncan, 2020). GPER receptors are also widely distributed in brain, and most concentrated in hippocampus and amygdala (Hadjimarkou and Vasudevan, 2018).

There is vast preclinical literature documenting that estradiol is neuroprotective and neurotrophic, with anti-inflammatory and vasodilating effects, especially in brain regions responsible for higher-order cognitive functions, including hippocampus, cerebral cortex and striatum (Brann et al., 2007). Estrogen is a “systems biology regulator” (e.g., a factor that regulates the frequency, rate or extent of interrelated biological processes) of neuronal function and survival, supporting neuronal plasticity through genomic and non-genomic actions (McEwen et al., 1997; Brinton et al., 2015; Lai et al., 2017), increases in spinogenesis and synaptogenesis (Hara et al., 2015), cell proliferation (Goodman et al., 1996), and gene expression (Woolley and McEwen, 1992; Brinton, 2008; Yin et al., 2015). In adult female rats, synaptic density of hippocampal neurons in the CA1 region were correlated with estradiol during the 5-day estrous cycle, demonstrating that fluctuations in estradiol levels directly mediate short-term synaptic density (Woolley and McEwen, 1992).

Further, mechanistic analyses led to the discovery that estrogen functions as a master regulator of the brain bioenergetic system, acting as a critical signaling molecule involved in glucose uptake and metabolism, mitochondrial respiration, and ATP generation (Rettberg et al., 2014; Brinton et al., 2015). Since glucose is the primary physiological substrate for ATP generation in the brain, energy production is at risk if reduction in glucose metabolism occurs. Several lines of research show that estrogen plays a major role in regulating several mitochondrial pathways, and that loss of estrogen precipitates mitochondrial dysfunction (Brinton et al., 2015). Further, estrogen promotes aerobic glycolysis and the citric acid cycle (TCA) by increasing the activity of glycolytic and TCA enzymes (Rettberg et al., 2014), pyruvate dehydrogenase (PDH), and ATP synthase (Nilsen et al., 2007). As a result, the menopausal transition in female animals is marked by a bioenergetic deficit characterized by downregulation of glucose metabolic pathways, in particular glucose transporter 3 (GLUT3), pyruvate dehydrogenase 1 (PDH1), and oxidative phosphorylation (Yao et al., 2011, 2012; Ding et al., 2013; Yin et al., 2015). Concomitant reductions in CMRglc as detected by FDG-PET are apparent in clinical analyses of oophorectomized women as well as those undergoing spontaneous menopause (Mosconi et al., 2017a,b, 2018, 2021; Rahman et al., 2020). Glucose metabolic decline in brain is also found during the prodromal phase of AD (Mosconi, 2005) and can activate inflammatory processes involved in AD pathophysiology (Mishra and Brinton, 2018; Mishra et al., 2020; Wang et al., 2020a,b). Collectively, these studies implicate a shift in the bioenergetic system of the brain during the menopause transition as a trigger for Aβ deposition, along with increased fatty acid catabolism, and declines in mitochondrial activity and synaptic plasticity (Liu F. et al., 2008; Brinton, 2009; Yao and Brinton, 2012), which could serve as early initiating mechanisms for AD.

These data are consistent with evidence that 17β-estradiol promotes non-amyloidogenic processing by increasing secretion of amyloid precursor protein (APP) and decreasing Aβ production (Xu et al., 1998; Manthey et al., 2001; Nord et al., 2010). Estrogen has been shown to upregulate Aβ -degradation enzymes such as metalloproteinases-2 and -9 (Merlo and Sortino, 2012), neprilysin (Liang et al., 2010), and insulin-degrading enzyme (IDE) (Zhao et al., 2011). Further, 17β-estradiol reduces levels of both induced and naturally occurring hyperphosphorylated tau protein (Alvarez-de-la-Rosa et al., 2005; Liu X. A. et al., 2008). On the other hand, estrogen loss following oophorectomy increases inflammation, tau hyperphosphorylation, accumulation of Aβ plaques and Aβ-induced neurotoxicity in transgenic mouse models of AD (Li et al., 2000; Levin-Allerhand et al., 2002; Yue et al., 2005; Nilsen et al., 2006).

## Endogenous Estrogen Exposures

### Menopause

Physiologically, menopause represents the permanent cessation of ovulation and menstrual cycles. It is defined retrospectively, after 12 months of amenorrhea without obvious pathologic cause. Menopause occurs in stages (Santoro, 2005). The early menopause transition is associated with lower ovarian inhibin secretion, which in turn, reduces the restraint on both the hypothalamus and pituitary, resulting in elevated follicle-stimulating hormone (FSH) and luteinizing hormone (LH) secretion, while ovarian estradiol secretion is normal or at times, elevated (Santoro, 2005). The late menopause transition (perimenopause) is characterized by periods of estrogen withdrawal, with fewer ovulatory cycles and prolonged hypogonadism, which ultimately lead to the final menstrual period (FMP) and a menopause diagnosis (Santoro, 2005). Production of other hormones such as androgens and growth factors also decline during perimenopause (Harlow et al., 2012). Five to 10 years after the FMP, hormonal levels become more stable with elevated gonadotropin secretion (Hall and Gill, 2001).

All women go through menopause in their lives, either through a natural midlife aging process or via surgical or pharmacological intervention. While the average lifespan has been steadily increasing over the past century, the average age at which reproductive senescence occurs has remained relatively constant between 45 and 55 years of age. Therefore, including the prepubescent years, women live at least a third of their lives in a hypogonadism state, and that number increases to up to half for women with surgical menopause.

One in every eight women in the U.S. undergo surgical menopause in their lives (Huo et al., 2021). In most cases, premature menopause results from the removal of both ovaries (bilateral oophorectomy with or without hysterectomy) or the removal of ovaries and fallopian tubes (salpingo-oophorectomy) before menopause, which lead to an abrupt cessation of ovarian estrogen production. Removal of only one ovary (unilateral oophorectomy) does not cause menopause, though several studies indicate a younger age at menopause compared to women who retain both ovaries (Rosendahl et al., 2017). Removal of the uterus alone (hysterectomy without oophorectomy) can reduce ovarian estrogen release by disturbing blood flow to the ovaries, thus indirectly influencing the onset of menopause. Certain drugs and radiation therapies can also damage the ovaries and prompt menopause.

Spontaneous menopause is a normal physiological event without long-term adverse effects for the majority of women (Monteleone et al., 2018). However, as high as 80% of women are vulnerable to the neurological shifts that can occur during the transition, experiencing an increased risk of neurological and psychiatric disorders including depression, anxiety, and dementia later in life (Brinton et al., 2015).

#### Surgical Menopause and Alzheimer’s Disease Risk

Oophorectomies are frequently performed for benign (non-cancerous) diseases, most commonly for recurrent ovarian cysts, endometriosis, chronic pelvic pain and also at the time of hysterectomy for fibroids and heavy vaginal bleeding (Hickey et al., 2010). Other common indications include removal of gynecological cancers or risk reduction treatment in women with an inherited increased chance of ovarian cancer as due to gene mutations such as BRCA1, BRCA2 or HNPCC (Rebbeck et al., 2009), or those with a strong family history of ovarian cancer (Parker et al., 2007).

These procedures are considered low-risk surgeries. However, abrupt discontinuation of ovarian function in premenopausal women is associated with more severe health consequences than spontaneous menopause, including a sudden and more severe onset of menopausal symptoms, especially hot flashes and vaginal dryness, and an increased risk of coronary artery disease, stroke, osteoporosis, and sexual dysfunction (Harlow et al., 2012). Surgical menopause has also been linked to a higher risk of depression and Parkinson’s disease (Rocca et al., 2008a,b). There is also evidence that sudden estrogen depletion following surgical menopause affects cognition and AD risk, as reviewed below and in Table 1.

**TABLE 1:** Effects of surgical menopause on cognitive function and AD risk.

References	Location	Type of study	Surgical menopause cases, *N*	Surgical procedure type	Controls, *N*	Age at menopause, mean (SD)	Follow up	Hormone therapy (HT) use	Endpoints	Primary and secondary outcomes
Sherwin, 1988	Canada	Randomized placebo controlled trial	40	Hysterectomy with bilateral oophorectomy	10	Surgical menopause group 45 (n.a.) years; control group: 37 (n.a.) years	4-, 5-, and 8-month postoperative follow-up	Climacteron, Delestrogen, Delatestryl, or placebo	Change in cognitive performance	• Oophorectomized women exhibited lower performance on all cognitive measures vs. non-oophorectomized women. • All HT formulations mitigated the decline in global cognition
Sherwin and Phillips, 1990	Canada	Randomized placebo controlled trial	12	Bilateral oophorectomy	n.a.	47 (n.a.) years	2 months	10 mg intramuscular estradiol valerate or placebo	Change in cognitive performance	• Oophorectomized women not taking HT exhibited lower performance on a paired-associates test vs. oophorectomized women taking HT. • HT use improved immediate and delayed logical memory scores over time
Phillips and Sherwin, 1992	Canada	Randomized placebo controlled trial	19	Bilateral oophorectomy	n.a.	48(5) years	2-month postoperative follow-up	10 mg intramuscular estradiol valerate injections or placebo	Change in cognitive performance	Oophorectomized women not taking HT exhibited lower performance on delayed paired-associates test vs. oophorectomized women taking HT. HT use improved immediate logical memory scores over time
Szklo et al., 1996	United States	Observational, longitudinal	1,088	Bilateral oophorectomy	5,022	Current HT users: 44(0.20) years; Past HT users: 45(0.20) years; Never users: 46(0.10) years; controls (n.a.)	3 years	Conjugated estrogen, or conjugated estrogen plus medroxyprogesterone	Change in cognitive performance	• Oophorectomized women aged 48–57 taking HT exhibited higher Controlled Oral Word Association scores vs. oophorectomized non-users. • No effects were observed in oophorectomized women aged 58–67
Nappi et al., 1999	Italy	Observational, cross-sectional	27	Hysterectomy with bilateral oophorectomy	76	Surgical menopause: 45(5) years; spontaneous menopause: 49(3) years	N/A	Women taking HT were excluded	Cognitive performance	• Oophorectomized women recalled fewer words during the serial learning test vs. spontaneous menopause. • Age at surgery was positively associated with verbal memory performance
Verghese et al., 2000	United States	Observational, cross-sectional	35	Bilateral oophorectomy	n.a.	HT users: 42(5) years; Non-users: 46(6) years	N/A	Conjugated estrogen, or conjugated estrogen plus medroxyprogesterone	Cognitive performance	Oophorectomized women taking HT exhibited higher scores on memory (BIMC), clock and block design tests than non-users
Farrag et al., 2002	Egypt	Observational, longitudinal	35	Hysterectomy and bilateral salpingo-oophorectomy	18	41(5) years	3- and 6- month post-operation	Women taking HT were excluded	Change in cognitive performance	• Oophorectomized women showed cognitive decline in MMSE and Wechsler Memory Scale subtests at follow-up visits. • Patients exhibiting >50% decline in estradiol had the most significant cognitive decline
McLay et al., 2003	United States	Observational, longitudinal	161	Not specified	200	43(9) years	Median 12.8 years	Women taking HT were excluded	Change in cognitive performance	No significant association between surgical menopause and cognitive performance
Rocca et al., 2007	United States	Observational, longitudinal	1,489	Unilateral or bilateral oophorectomy with or without hysterectomy	1,472	Not reported	Median 25–30 years	Not specified	Cognitive impairment and dementia incidence	• Unilateral and bilateral oophorectomy before age 49 increased risk of cognitive impairment or dementia. Risk increased with surgery at younger age. • HT mitigated this effect in women with bilateral oophorectomy
Ryan et al., 2009	France	Observational, longitudinal	186	Not specified	810	50(5) years	2- and 4- years	Transdermal estradiol with or without progesterone	Change in cognitive performance	No significant association between surgical menopause and cognitive performance
Phung et al., 2010	Denmark	Observational, longitudinal	215,444	Hysterectomy, Unilateral or Bilateral Oophorectomy	2,097,944	Hysterectomy: 48 (n.a.) years; Unilateral oophorectomy: 45 (n.a.) years; Bilateral oophorectomy: 57 (n.a.) years	16.6 years	Not specified	Dementia incidence	• Younger age at oophorectomy and hysterectomy was associated with early dementia. • Hysterectomy with bilateral oophorectomy had greatest incidence of dementia among 50–59 years old
Zhou et al., 2011	China	Observational, cross sectional	50	Unilateral oophorectomy with or without hysterectomy	50	43(3) years	N/A	Women taking HT were excluded	Cognitive performance	Oophorectomized women exhibited lower performance on immediate and delayed word recall vs. spontaneous menopause
Bove et al., 2014	United States	Observational, longitudinal	603	Not specified	1,281	Surgical menopause: 43(7) years; Spontaneous menopause: 49(5) years	Annually, up to 18 years	Not specified	Change in cognitive performance; AD incidence; brain autopsy samples	• Surgical menopause at a younger age was associated with steeper global cognitive decline. • Surgical menopause was associated with increased amyloid plaque burden but not with AD incidence. • HT initiated within 5 years of menopause was associated with decrease in global cognitive decline but not with AD pathology
Ryan et al., 2014	France	Observational, longitudinal	487	Bilateral oophorectomy	4,381	Surgical menopause: *n* = 136 age <40; *n* = 115 age 41–45; *n* = 152 age 46–50; *n* = 96 age > 50; Spontaneous menopause: *n* = 100 age <40; *n* = 366 age 41–45; *n* = 1,556 age 46–50; *n* = 1,820 age > 50	2-, 4-, and 7- years	Current and past users of transdermal estradiol, or unopposed estradiol	Change in cognitive performance and dementia incidence	• Oophorectomized women with younger age at surgical menopausal exhibited a 35% increased risk of global cognitive decline. • HT had negative effects on verbal fluency performance in this cohort. • No association with dementia risk at the 7-year follow up
Kurita et al., 2016	United States	Observational, longitudinal	123	Bilateral oophorectomy	803	Surgical menopause: 45(5) years; Spontaneous menopause: 50(6) years	3(1) years	Women taking HT were excluded	Change in cognitive performance	Oophorectomy before menopause was associated with visual and semantic memory decline vs. spontaneous menopause
Zeydan et al., 2019	United States	Observational, cross-sectional	23	Bilateral salpingo-oophorectomy	20	Surgical menopause: median 46 years; controls n.a.	N/A	Conjugated equine estrogen, conjugated equine estrogen with progestin	Cognitive performance; MRI and PiB PET biomarkers	• No differences in cognitive scores between surgical and spontaneous menopause groups. • Oophorectomized women exhibited smaller amygdala volume and parahippocampal-entorhinal cortex thickness, and lower fractional anisotropy vs. spontaneous menopause. • There were no effects on hippocampal volume and PiB uptake

Since the late 1980s, several reports indicated memory declines in women undergoing oophorectomy before menopause, which were later substantiated by large-scale studies indicating an almost doubled long-term risk of dementia in oophorectomized women (Rocca et al., 2007, 2014; Phung et al., 2010; Bove et al., 2014). Dementia risk is generally highest in the presence of bilateral oophorectomy, intermediate with unilateral oophorectomy, and lowest but significant in the presence of hysterectomy without oophorectomy (Yaffe et al., 1998; Hogervorst et al., 2000; LeBlanc et al., 2001; Rocca et al., 2007; Phung et al., 2010; Bove et al., 2014; Gilsanz et al., 2019). Bilateral oophorectomy also results in an abrupt drop in circulating levels of progesterone and testosterone, and in the disruption of the hypothalamic-pituitary-gonadal (HPG) axis (Morrison et al., 2006), which causes a sudden increased release of LH and FSH in turn (Rocca et al., 2018). Additionally, pre-menopausal oophorectomies have been linked to alterations in nervous system innervation with wide-ranging effects on the function of the vagus nerve, autonomic nervous system, and cardiovascular system, among others (Mercuro et al., 2000; Rocca et al., 2018). Dementia risk increases with younger age at time of surgery (Rocca et al., 2008b; Phung et al., 2010), which has also been associated with increased global AD burden at post-mortem, especially the load of neuritic amyloid plaques (Bove et al., 2014; Agca et al., 2020).

Surgical menopause has been linked to more severe consequences on cognitive function than spontaneous menopause, including lower performance in verbal learning, visual memory (Rocca et al., 2007), and delayed word recall tasks (Zhou et al., 2011). Decline in short-term verbal memory was more severe in women who had greater than 50% decline in serum estradiol levels following surgery (Nappi et al., 1999; Farrag et al., 2002). However, as discussed below, estrogen replacement therapy following surgery appears to mitigate cognitive changes when compared to placebo (Sherwin and Phillips, 1990; Henderson et al., 2005; Rocca et al., 2007, 2010; Whitmer et al., 2011; Shao et al., 2012), suggesting that post-operative estrogen therapy may have a neuroprotective effect.

#### Spontaneous Menopause and Alzheimer’s Disease Risk

Given the importance of estrogen for brain function, it is not surprising that complaints of a decline in memory, attention and concentration are common during the menopause transition (Gold et al., 2000). These complaints are, however, self-reported and unlikely to result in objective, measurable impairment, thus often falling under the diagnostic category of subjective cognitive decline (SCD). Although SCD is observed in normal aging and in some psychiatric, neurological, and medical conditions other than AD, current evidence suggests that people ages 60 and older experiencing SCD may be at higher risk for MCI and dementia (Jessen et al., 2014). Notably, SCD is more common, and may be more predictive of later cognitive dysfunction, in women ages 65 and above (Pérès et al., 2011).

Whether menopause-related cognitive complaints can be confirmed objectively is a topic of debate (Mitchell and Woods, 2011; Weber et al., 2012). Some studies indicate measurable, yet modest declines in verbal episodic memory on delayed recall tests, or lack of improvement in verbal memory and processing speed with repeated testing (Fuh et al., 2006; Greendale et al., 2009, 2011; Bromberger et al., 2010; Berent-Spillson et al., 2012; Epperson et al., 2013; Weber et al., 2013). These findings were more likely restricted to the perimenopausal and early postmenopausal stages, and were independent of non-cognitive menopausal symptoms such as anxiety, disturbed sleep, and mood symptoms (Greendale et al., 2010).

In some studies, SCD in perimenopausal women was associated with changes in working memory and complex attention rather than verbal episodic learning or memory (Weber et al., 2012), suggesting that cognitive operations demanding higher effort may lead to women’s perception of cognitive difficulties. Importantly, memory declines during perimenopause range from moderate to subtle, and performance tends to rebound to premenopausal levels after the menopause diagnosis (Greendale et al., 2009; Weber et al., 2013). Overall, clinical data suggest that negative effects of menopause on cognitive performance are transient and generally limited to the perimenopausal stage. It is unknown whether these memory fluctuations are predictive of cognitive impairment in later life.

Novel research using AD biomarkers provides important insight into the neurological effects of menopause and its effects on cognition and AD risk. While the preponderance of biomarker studies has been carried out in women who had already transitioned through the menopause, recent investigations targeting women of perimenopausal age demonstrated significant associations between menopause and biomarker indicators of increased AD risk. For instance, smaller medial temporal lobe volume was reported in surgical menopausal cases as compared to spontaneous menopause (Zeydan et al., 2019) and in recently postmenopausal women with SCD (Conley et al., 2020). Presence of night sweats was associated with a higher burden of WM hyperintensities (Thurston et al., 2016), indicating a link between vasomotor symptoms and cerebral small vessel disease, a risk factor for stroke and dementia (Debette and Markus, 2010).

**FIGURE 1:**
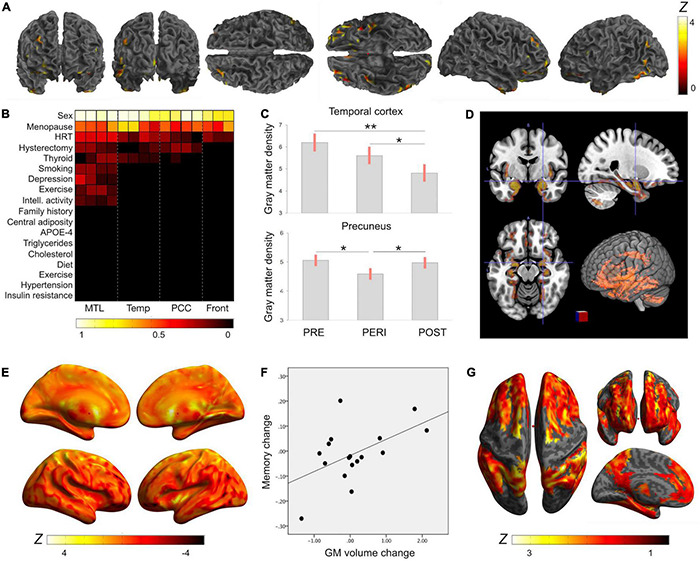
Reproductive history effects on gray matter volume. Summary of MRI studies indicating associations of reproductive history indicators and MRI-derived gray matter (GM) volume: **(A)** Surface renderings of statistical parametric maps displaying areas of lower GM volume in midlife women as compared to age-controlled men (Rahman et al., 2020). Results are displayed on a color-coded scale with corresponding *Z* values, where *Z* > 3 corresponds to *p* < 0.05, corrected for multiple comparisons. **(B)** LASSO regression models indicate that midlife sex differences in GM volume were driven by menopause status, followed by use of hormonal replacement therapy (HT) and hysterectomy status (Rahman et al., 2020). Coefficients from LASSO regressions ranking multiple exposures are displayed on a color-coded scale such that coefficients >0.5 correspond to *p* < 0.05. **(C)** Among midlife women, GM density was highest in premenopausal women (PRE), intermediate in perimenopausal women (PERI), and lowest in postmenopausal women (POST), adjusting by age and intracranial volume (Mosconi et al., 2017b,^2021^); Plots display the mean, covariate-adjusted GM density (SE) in temporal cortex and precuneus of PRE, PERI and POST groups; **p < 0.001, *p < 0.01. **(D)** Brain regions undergoing GM volume changes as a result of the menopause transition exhibit substantial anatomical overlap with the brain estrogen network, including medial temporal lobe, insula, anterior and posterior cingulate, precuneus, parieto-temporal and frontal cortices (Rahman et al., 2020). Statistical parametric maps showing 3D GM volume differences between PRE, PERI and POST groups are superimposed on a standardized T1-MRI image at *p* < 0.05. **(E)** In longitudinal studies, GM volume declined in temporal regions of postmenopausal groups (negative values), but showed recovery in precuneus (positive values) (Mosconi et al., 2018, 2021). Surface renderings display significant GM volume changes post-menopause, which are represented on a color-coded scale with corresponding *Z* values, where *Z* > 3 and *Z* < −3 correspond to *p* < 0.05. **(F)** GM volume changes in precuneus correlated with memory changes among postmenopausal women (Mosconi et al., 2021). **(G)** GM volume is influenced by additional reproductive history events such as a longer reproductive span and exogenous estrogen exposure (Schelbaum et al., 2021). Statistical parametric maps (SPM) displaying brain regions showing significant associations between longer reproductive spans and GM volume. Effects are represented on a color-coded scale with corresponding *Z* values, where *Z* > 3 corresponds to *p* < 0.05.

More direct evidence that the menopause transition is associated with AD risk comes from multi-modality brain imaging studies reporting emergence of AD endophenotypes in midlife women carrying risk factors for AD, such as APOE-4 genotype and a family history of late-onset AD (Figure 1). In these studies, perimenopausal and postmenopausal women exhibited higher Aβ load, lower CMRglc, and lower GM and WM volume as compared to premenopausal women and to age-controlled men, independent of age and midlife health indicators (Mosconi et al., 2017a,b, 2018, 2021; Rahman et al., 2020; Figures 1A–C). AD biomarker effects involved brain regions vulnerable to AD, such as posterior cingulate, precuneus, parieto-temporal, medial temporal, and frontal cortices (Figure 1D). These regions exhibit considerable overlap with the brain estrogen network (Brinton et al., 2015; Figure 1D), further highlighting the connection between endocrine aging and cognitive aging in women. Biomarker effects correlated with menopause status being lowest premenopause, intermediate in perimenopause, and greatest postmenopause (Mosconi et al., 2017a,b, 2018, 2021; Rahman et al., 2020). Additionally, perimenopausal and postmenopausal women, especially those positive for APOE-4 genotype, exhibited the highest Aβ burden (Mosconi et al., 2017b,^2021^), supporting the notion that APOE-4 genotype exacerbates AD-related brain changes in women (Riedel et al., 2016), with onset in perimenopause. While menopause effects on Aβ deposition were overall mild, the earlier onset and longer exposure to Aβ pathology could help account for the higher prevalence of AD in women.

Longitudinal exams indicate that biomarker abnormalities related to the menopause transition were progressive over a 3 year period (Mosconi et al., 2018). However, some brain regions showed stabilization and in some cases, recovery later into menopause (Mosconi et al., 2021). For example, GM volumes in temporal regions and precuneus declined in perimenopausal and recently postmenopausal women (Mosconi et al., 2018), but increased in late postmenopause (Mosconi et al., 2021; Figure 1E). Additionally, in spite of WM volume declines, fractional anisotropy (FA), as derived using Diffusion Tensor Imaging (DTI), remained broadly stable during the menopause transition (Mosconi et al., 2021). Since FA is a marker of WM integrity and structural connectivity, these data suggest preserved connectivity of smaller WM tracts (Mosconi et al., 2021). Moreover, cerebral blood flow (CBF), as measured by means of Arterial Spin Labeling (ASL), was higher in the postmenopausal group as compared to premenopausal controls and to age-controlled men, and so were ATP levels measured via ^31^Phosphorus Magnetic Resonance Spectroscopy (^31^P-MRS) (Mosconi et al., 2021). GM volume recovery and brain ATP production post-menopause correlated with cognitive performance (Mosconi et al., 2021), supporting the clinical findings above. On the other hand, biomarker recovery was attenuated in perimenopausal and postmenopausal APOE-4 carriers, who also exhibited the highest Aβ load (Mosconi et al., 2021).

Overall, this preliminary data suggests brain adaptation to the hypo-estrogenic postmenopausal state, at least in some women, consistent with findings of system-biology adaptations in response to estrogen decline in aging female animals (Wang et al., 2020b). This may account for the easing of menopausal symptoms observed in late postmenopausal women (Monteleone et al., 2018). Studies comparing women at high vs low risk for AD are needed to determine whether the hypothesized brain recovery is comprised in presence of an AD predisposition.

## Other Reproductive Health Indicators

**FIGURE 2:**
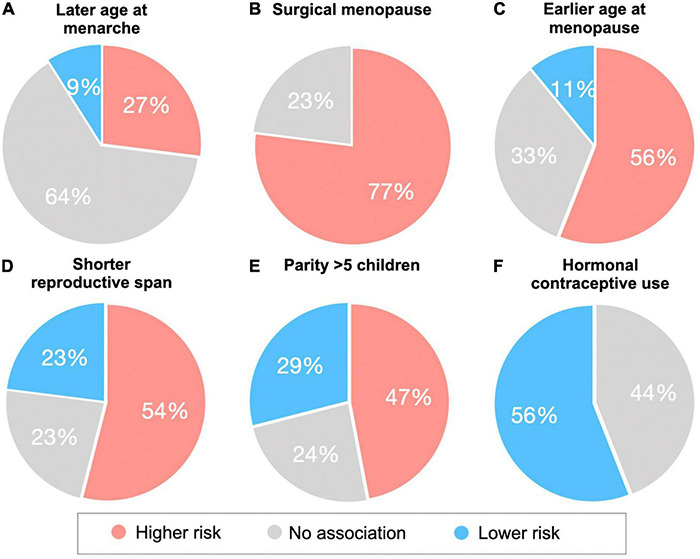
Associations of reproductive health indicators with cognitive aging, AD biomarkers and AD risk. Reproductive health indicators are **(A)** age at menarche, **(B)** type of menopause (surgical vs spontaneous), **(C)** age at menopause, **(D)** reproductive span, **(E)** grandparity vs nulliparity, **(F)** hormonal contraceptive use. Pie charts summarize the percentage of studies reporting effects of reproductive health indicators and endpoints (including AD or dementia incidence, cognitive performance, and AD biomarkers) in terms of higher, lower, or null AD risk. For example, in panel **(C)**, 56% of studies report associations between earlier age at menopause and higher AD risk.

Even before menopause, the level of estrogen in the female brain fluctuates based on several reproductive events and hormone treatments. Reproductive history is an important modifier of lifetime estrogen exposure and likely of cognitive aging. As reviewed below, there has been increasing interest in determining whether estrogen exposure over the female lifespan is protective against AD. Key findings are summarized in Table 2 and Figure 2.

**TABLE 2:** Effects of reproductive history factors on cognitive function and AD risk.

References	Location	Type of study	Study population	Inclusion of surgical menopause cases	Age at cognitive assessment, mean (SD)	Follow up visits	Exposures	Endpoints	Primary outcomes
Geerlings et al., 2001	Netherlands	Observational, longitudinal	3,601 cognitively normal women aged over 60 years	2,737 women with spontaneous menopause; 865 women with surgical menopause	70(9) years	6 years; range 0–9 years	Age at menarche, age at menopause, reproductive span	Dementia and AD incidence	• Longer reproductive span and later age at natural menopause were associated with dementia and AD incidence in APOE-4 carriers. • Age at menarche was not associated with dementia or AD.
Rasgon et al., 2005a	Sweden	Observational, cross-sectional	5,844 women aged 65–84 years (1,111 with cognitive impairment, 4,733 controls)	Not specified	Cognitively Impaired: 75(6) years; Controls: 72(5) years	N/A	Age at menarche, age at menopause, reproductive span, total length of estrogen exposure (reproductive span + HT duration years), number of childbirths, HT use and duration	Difference between dementia patients and controls	• Menarche before age 12 or after age 14, and shorter reproductive span were associated with dementia. • Dementia patients had lower age at menopause than controls • Women with ≥ 5 children were more likely to have dementia compared to women with 1–2 children. • HT use was more common in controls than in dementia patients
Colucci et al., 2006	Italy	Observational, cross-sectional	405 women over 65 years (204 probable AD patients, 201 controls)	AD patients: 21% surgical menopause; Controls: 13% surgical menopause	AD patients: 75(7) years; Controls: 74(6) years	15 months	Age at menarche, age at menopause, reproductive span, number of pregnancies and miscarriages, surgical menopause, breast pathology, HT use	Difference between AD patients and controls	• No differences in age at menarche, age at menopause, reproductive span, number of miscarriages, or breast pathology between AD patients and controls. • Women with AD were more likely to have 3 or more children compared to controls.
Ryan et al., 2009	France	Observational, longitudinal	996 cognitively normal women aged 65–94 years	810 women with spontaneous menopause; 186 women with surgical menopause	73(6) years	2- and 4 years	Age at menarche, age at menopause, reproductive span, parity (number of children), age at first birth, HT use, contraceptive use	Cognitive performance; Dementia incidence	• Longer reproductive span was associated with higher verbal fluency scores but not with dementia incidence • Younger age at menarche was associated with higher visual memory and psychomotor speed. • Women who had their first birth between ages 21–29 scored higher on verbal fluency, visual memory, and psychomotor speed tests vs. women who gave birth before age 21. • Current HT use, but not past, was associated with higher visual memory scores vs. never users. • No associations between contraceptive use or surgical menopause with cognitive performance or dementia incidence.
Heys et al., 2011	China	Observational, longitudinal	8,685 cognitively normal women aged 50–95 years	Women with hysterectomy or oophorectomy were excluded	60(7) years	1–3 years	Reproductive span, parity (number of children), age at first birth, time spent breastfeeding	Change in cognitive performance	• Longer reproductive span, lower parity, and less time spent breastfeeding were associated with higher cognitive performance on word recall and MMSE. • Older age at first birth was associated with lower cognitive performance on delayed word recall and MMSE.
Fox et al., 2013	England	Observational, cross-sectional	89 women aged 70–100 years (38 AD patients, 51 controls)	32 women with surgical menopause	Dementia: 86(6) years; Controls: 77 (7) years	N/A	Age at menarche, age at menopause, reproductive span, total length of estrogen exposure (reproductive span and HT duration years minus time spent breastfeeding), time spent pregnant, parity, age at first birth; time spent breastfeeding, number of menstrual cycles, HT use, contraceptive use	Difference between AD patients and controls	• Controls exhibited longer cumulative estrogen exposure, first birth after age 21, and more months spent pregnant vs. AD patients. • No associations between reproductive span, age at menarche, age at menopause, parity, contraceptive use, HT use, or regularity of menstrual cycles with AD status
Prince et al., 2018	Latin America, China	Observational, longitudinal	6,854 cognitively normal women over 65 years in Latin America and China	Not specified	74(7) years	3–5 years	Age at menarche, age at menopause, reproductive span, index of cumulative endogenous estrogen exposure parity (number of live births), age at first birth, premature ovarian failure	Dementia incidence	• Greater parity was associated with increased dementia incidence. • No association between age at menarche, age at menopause, reproductive span, index of cumulative endogenous estrogen exposure, age at first birth, or premature ovarian failure with dementia incidence
Gilsanz et al., 2019	United States	Observational, longitudinal	6,137 cognitively normal women, mean age 51(4) years at baseline	4,047 women with spontaneous menopause;2,090 women with hysterectomy	77(5) years	9(6) years; range 0–22 years	Age at menarche, age at menopause, reproductive span, hysterectomy status	Dementia and AD incidence	Later age at menarche, menopause before 47 years, reproductive spans shorter than 34 years, and having had a hysterectomy were associated with higher dementia incidence
Matyi et al., 2019	United States	Observational, longitudinal	2,114 cognitively normal women over 65 years	Not specified	75(7) years	Triennial visits for 12 years	Lifetime endogenous estrogen exposure (reproductive span minus time spent breastfeeding), HT use	Change in cognitive performance	• Longer endogenous estrogen exposure was associated with higher modified MMSE (3MS) scores. • Longer use of HT and initiation of HT within 5 years of menopause were associated with higher 3MS scores.
Najar et al., 2020	Sweden	Observational, longitudinal	1,364 cognitively normal women, mean age 53(6) years at baseline	Women with hysterectomy or oophorectomy were excluded	80(8) years	27(10) years	Age at menarche, age at menopause, reproductive span, number of pregnancies, time spent breastfeeding, HT use, contraceptive use	Dementia and AD incidence	• Later age at menopause and longer reproductive span were associated with increased risk of dementia and AD. • No associations between age at menarche, number of pregnancies, time spent breastfeeding, HT use, or contraceptive use with dementia or AD incidence.
Song et al., 2020	China	Observational, longitudinal	9,656 women aged 45–74 years at baseline	8,222 women with spontaneous menopause; 1,324 with surgical menopause	78(7) years	Range 5–23 years	Age at menarche, age at menopause, reproductive span, number of children, age at first birth, HT use, contraceptive use and duration	• Cognitive performance	Older age at menopause, longer reproductive span, short-term contraceptive use, and HT use were positively associated with SM-MMSE performance. • Women with ≥ 5 children had lower cognitive scores vs. 1–2 children. • No association between age at menarche, age at first birth, and type of menopause with cognitive performance
Najar et al., 2021	Sweden	Observational, longitudinal	75 women aged 46–60 years at baseline, free of dementia	Women with hysterectomy or oophorectomy were excluded	74 years; range 70–85 years	20(4) years	Age at menarche, age at menopause, reproductive span	CSF Aβ42, Aβ42/Aβ40, hyperphosphorylated tau (P-Tau), total tau (T-Tau)	• Earlier age at menarche and longer reproductive span were associated with higher P-Tau and lower Aβ42/Aβ40. • Longer reproductive period was associated with lower Aβ42. • No associations between age at menopause and CSF biomarkers.
Schelbaum et al., 2021	United States	Observational, cross-sectional	99 cognitively normal women aged 40–65 years	36 women with spontaneous menopause; 13 women with surgical menopause	52(6) years	N/A	Age at menarche, age at menopause, reproductive span, number of pregnancies and children, HT use, contraceptive use, hysterectomy status	Cognitive performance; gray matter volume (GMV) on MRI	• Longer reproductive span, number of children and pregnancies, HT use, and oral contraceptive use were associated with larger GMV in regions vulnerable to cognitive aging and AD. • No association between age at menarche, age at menopause, or hysterectomy status with GMV.

### Age at Menarche

The age at which a woman enters menarche (puberty) has gained attention for a possible relationship with cognition in later life due to higher estrogen levels, and thus longer estrogen exposure, when menarche occurs at a younger age (Bernstein et al., 1991). However, as shown in Figure 2A, the majority of studies so far indicate null associations between age at menarche and cognitive impairment or AD risk (Geerlings et al., 2001; Henderson et al., 2003; Colucci et al., 2006; Fox et al., 2013; Prince et al., 2018; Najar et al., 2020; Song et al., 2020). On the other hand, some studies reported that a younger age at menarche was associated with better visual memory and psychomotor speed (Ryan et al., 2009), and reduced risk of dementia or AD in later life (Rasgon et al., 2005a; Gilsanz et al., 2019). Additionally, the Gothenburg H70 Birth Cohort study reported associations between a younger age at menarche and lower CSF Aβ_42/40_ ratio and higher hyperphosphorylated tau levels among older postmenopausal women free of dementia (Najar et al., 2021).

### Age at Menopause

Given findings of neuroprotective effects of estrogen, it is plausible that a later age at menopause would enable longer overall exposure to estrogen, and supposedly better cognitive function. The majority of studies so far indicate positive outcomes with a later age at menopause (Figures 2B,C). As mentioned above, a younger age at surgical menopause has been linked to higher risk of AD (Bove et al., 2014), dementia (Rocca et al., 2008b; Phung et al., 2010), and cognitive decline (Rocca et al., 2007; Zhou et al., 2011). Although data on spontaneous menopause are less consistent, some studies also report better cognitive performance (McLay et al., 2003; Georgakis et al., 2016a), reduced risk of cognitive impairment (Song et al., 2020) or dementia (Rasgon et al., 2005a; Gilsanz et al., 2019), and a later age at AD onset (Sobow and Kloszewska, 2004) when menopause occurs later in life. In the Kaiser Permanente cohort, women who underwent spontaneous menopause between the ages of 31–40 and 41–45 years had a 20% and 29% increased risk of dementia, respectively, when compared to those who were aged 51–55 years at the time of menopause (Gilsanz et al., 2019). On the other hand, two studies reported an increased incidence of dementia and AD with an older age at menopause for specific study populations (Geerlings et al., 2001; Najar et al., 2020). In the Rotterdam study, this association was found only in women carrying at least one copy of the APOE-4 allele (Geerlings et al., 2001). In the Gothenburg H70 Birth Cohort study, the association was only significant in women who had dementia onset after 75 years of age, with the strongest occurrence after 85 years of age (Najar et al., 2020), whereas no association with AD was observed before age 85 or with APOE-4 status (Najar et al., 2020). Null associations have also been reported (Colucci et al., 2006; Fox et al., 2013; Prince et al., 2018; Najar et al., 2021).

### Reproductive Span

While both age at menarche and age at menopause mark the duration of a woman’s lifetime exposure to endogenous estrogen, examination of the time in between these two events – the reproductive span – appears to be a stronger predictor of later life cognition. Given the neuroprotective effects of estrogen, it is plausible that a longer reproductive span would be correlated with better cognitive outcomes in later life. The length of this window has been associated with reduced risk of several diseases in postmenopause, such as cardiovascular disease and depression (Georgakis et al., 2016b; Ley et al., 2017). However, the relationship between reproductive span and dementia risk is less clear (Table 2 and Figure 2D).

Several studies have found that women with longer reproductive periods, especially in presence of older age at menopause, have better cognitive outcomes and lower risk of dementia or cognitive decline (Rasgon et al., 2005a; Fox et al., 2013; Georgakis et al., 2016a; Gilsanz et al., 2019). In the Kaiser Permanente study, a reproductive span less than 34.4 years was associated with a 20% increased risk of dementia compared to those with a reproductive span of at least 34.4 years, and when comparing the groups of those with the shortest and longest reproductive span, 14–20 years and 39–44 years, respectively, there was a 55% increased risk of dementia associated with a shorter reproductive span (Gilsanz et al., 2019).

However, other studies observed no associations between reproductive span and dementia risk (Georgakis et al., 2016a; Prince et al., 2018) or verbal memory (Henderson et al., 2003), while two studies reported associations between longer reproductive periods and higher dementia risk among older women (Geerlings et al., 2001; Najar et al., 2020). In a follow-up analysis of the Gothenburg H70 Birth Cohort, longer reproductive span also correlated with lower CSF Aβ_42/40_ and higher phosphorylated tau levels in a subset of postmenopausal women ages 70–85 years (Najar et al., 2021). The authors offer that women who undergo menopause at an older age have higher estrogen levels later in life, when AD-related pathologies arise. This is consistent with the “healthy cell bias” theory, which postulates that while estrogen is neuroprotective in healthy cells, it can be neurotoxic in damaged cells (Brinton, 2008). Thus, the timing of endogenous estrogen exposure likely contributes to the positive or negative impact of estrogen on neuronal health. However, as all women in the Gothenburg study remained free of dementia despite the biomarker data, the functional significance of the associations is unclear. In fact, these correlations may suggest a protective effect of reproductive span instead – analogous to the way that more years of education correlate with higher AD pathology burden in non-demented elderly, which are interpreted as reflecting greater cognitive reserve (Barulli and Stern, 2013). For context, over 40% of dementia free elderly exhibit AD biomarkers in pathological range (Roberts et al., 2018), with Aβ positivity increasing from ∼10% at age 50 to an average of 44% or higher at age 90 in cognitively normal elderly (Jansen et al., 2015).

**FIGURE 3:**
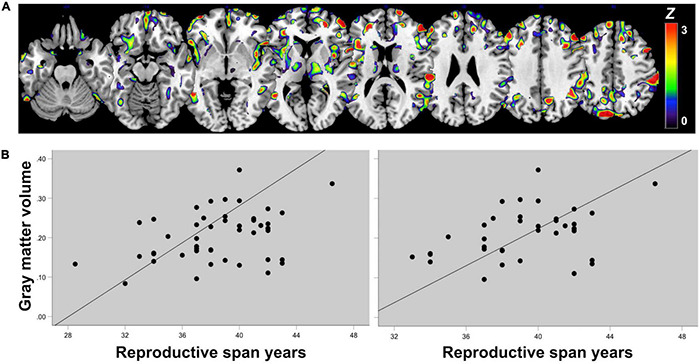
Associations of reproductive span with regional gray matter volume in midlife. **(A)** Statistical parametric maps displaying significant associations between reproductive span years and GM volume are superimposed on a standardizedT1-MRI image and represented on a color-coded scale with corresponding *Z* values, at *p* < 0.05 corrected for multiple comparisons. **(B)** Scatterplots of associations between reproductive span and GM volume in superior parietal and precuneus in (left) the entire postmenopausal cohort and (right) among women who had undergone spontaneous menopause, thus excluding surgical menopause cases. Figures are modified from results included in Schelbaum et al. (2021).

In a recent brain MRI study of midlife women, longer reproductive span was associated with larger superior parietal and precuneus GM volume, independent of age, APOE-4 status, type of menopause (surgical vs spontaneous) midlife health indicators, and use of menopausal HT (Schelbaum et al., 2021; Figure 3). GM volume is a proxy for cognitive reserve, with larger volume indicating less neuronal aging (Jack et al., 2013). Therefore, MRI findings suggest a beneficial effect of longer endogenous estrogen exposure, at least in midlife. Since the pre-symptomatic phase of AD corresponds with midlife years (Sperling et al., 2011), and endogenous estrogen exposures occur before or concomitant with menopause, simultaneous examination of reproductive span and biomarker data avoids possible effects of attrition, recall, and survival bias. In fact, these results are consistent with epidemiological studies of younger women with a narrower window of age at menopause (Fox et al., 2013; Gilsanz et al., 2019). The two studies reporting negative effects of a longer reproductive span on AD risk instead focused on older postmenopausal women over 70 (Geerlings et al., 2001; Najar et al., 2020), excluded patients with hysterectomy or oophorectomy (Geerlings et al., 2001; Najar et al., 2020), and in some cases did not assess use of hormonal therapy (Geerlings et al., 2001), or reported effects only in APOE-4 carriers (Geerlings et al., 2001), which could account for the mixed findings. Further comparison across studies is made difficult by substantial heterogeneity of the study cohorts, including differences in geographical areas, ethnicities, socio-economic backgrounds, and overall medical health, as well as different clinical criteria for AD and dementia diagnosis, and variable cognitive batteries across studies.

### Parity, Number of Children and Pregnancies

Pregnancy is a female-specific reproductive event that induces profound changes in the levels of endogenous estrogen, with significant effects on brain structure and function (de Lange et al., 2020). Higher levels of estradiol are observed during pregnancy, up to 300 times the normal circulating levels (Deems and Leuner, 2020), which may have beneficial effects in terms of cumulative estrogen exposure throughout a woman’s life. However, the effects of pregnancy on the brain are complex and more rigorous work is needed to elucidate how these biological mechanisms impact cognitive aging. For instance, parous women have lower levels of circulating estrogen compared to nulliparous women, with this difference persisting into menopause (Bernstein et al., 1985). On the other hand, parity has positive effects on the brain’s long-term sensitivity to estrogen via effects on ER expression or functioning as evidenced by findings that parous rats have higher numbers of ERα positive cells than age-matched, nulliparous controls (Byrnes et al., 2009). There is also evidence that parity renders the human brain more responsive to estrogen in older age, which might contribute to favorable brain aging trajectories (de Lange et al., 2019).

In studies investigating the long-term association between pregnancy-related factors and risk of cognitive impairment in later life, associations are mixed, as summarized in Table 3.

**TABLE 3:** Pregnancy-related factors, cognitive function and dementia risk.

References	Location	Study population	Inclusion of nulliparous women	Follow Up Visits	Parity-related exposures	Mean age (SD), years	Endpoints	Primary outcomes
Ptok et al., 2002	Germany	295 women (106 with AD, 117 with depression, 72 controls)	Yes	N/A	Parity (parous vs nulliparous); number of children	AD: 77(10); depression: 71(8); Control: 72(11)	Difference between AD patients and controls	• Women with AD were more likely to be parous than nulliparous. • Number of children was not associated with AD diagnosis
Henderson et al., 2003	Australia	326 healthy premenopausal women	Yes	8 years	Parity (parous vs nulliparous); number of pregnancies	57(3) years	Change in cognitive performance	Parity was positively associated with verbal memory
McLay et al., 2003	United States	361 Caucasian and African American women with and without dementia	Yes	13 years	Number of live births, oral contraceptive usage	63(14) years	Change in cognitive performance	• Parity was associated with cognitive decline. • Oral contraceptive use was not associated with cognitive function
Dunkin et al., 2005	United States	17 healthy postmenopausal women	Yes	10 weeks	Number of childbirths	57(7) years	Change in cognitive performance	No associations between parity and cognitive performance
Rasgon et al., 2005a	Sweden	5,844 women (1,111 with cognitive impairment, 4,733 controls)	Yes	N/A	Number of children	Cognitively Impaired: 75(6) years; Control: 72(5) years	Difference between dementia patients and controls	Women with dementia were more likely to have ≥ 5 children compared to controls.
Colucci et al., 2006	Italy	405 women (204 with probable AD, aged 75(7) years, 201 controls aged 74(6) controls)	Yes	15 months	Number of pregnancies	AD: 75(7) years; Control: 74(6) years	Difference between AD patients and controls	• AD patients had more pregnancies than controls. • 3 or more pregnancies were associated with a younger age at onset of AD. • No association between number of pregnancies and change in MMSE scores
Corbo et al., 2007	Italy	176 women with sporadic AD	Yes	N/A	Number of children	77(7) years	Association with age at onset of AD	• Women with children had a younger age at AD onset compared to nulliparous women. • No associations between number of children and age at onset. • Age at AD onset was younger in APOE4 negative parous women.
Beeri et al., 2009	United States	73 AD patients (42 women and 31 men) confirmed at autopsy	Yes	N/A	Number of children	Women: 86(11) years; Men: 73(9) years	Association with AD pathology	• Women who had more children had greater AD neuropathology and neuritic plaques vs. nulliparous women. • No associations between number of children and AD pathology in men.
Ryan et al., 2009	France	996 cognitively normal women	Yes	2-, 4- years	Parity (parous vs nulliparous), age at first birth, oral contraceptive use	73(6) years	Cognitive performance; Dementia incidence	• Women who gave birth before age 21 had lower MMSE scores, visual memory scores, and psychomotor speed vs. those with first birth between 21 and 29 years. • No associations between parity and oral contraceptive with cognitive performance. • No association between parity, age at first birth, or oral contraceptive use with dementia incidence.
Heys et al., 2011	China	8,685 cognitively normal women	No	1–3 years	Number of children, age at first birth, breastfeeding duration	60(7) years	Change in cognitive performance	• Lower parity and less time spent breastfeeding were associated with higher cognitive performance on word recall and MMSE. • Older age at first birth was associated with lower cognitive performance on delayed word recall and MMSE.
Fox et al., 2018	England	95 women (39 with dementia, 56 controls)	Yes	N/A	Parity (nulliparous vs parous), age at first birth, cumulative months pregnant, cumulative months breastfeeding	Dementia: 86(6) years; Control: 77(7) years	Difference between AD patients and controls	• AD patients had fewer cumulative months pregnant compared to controls. • Controls had a greater number of first trimesters than women with AD.
Bae et al., 2020	Europe/Asia	7,010 dementia free women	Yes	5(3) years	Number of childbirths or children	72(8) years	Dementia or AD incidence	• Parity was not associated with AD incidence. • All-cause dementia and non-AD dementia were associated with increasing parity
de Lange et al., 2020	Europe	19,787 cognitively normal women	Yes	N/A	Number of live childbirths, age at first birth, years since last birth	64(7) years	MRI-derived brain age	Increasing parity was associated with younger ‘brain age’ on MRI
Jung et al., 2020	Korea	237 women (89 with and 148 without MCI)	Yes	N/A	Parity (nulliparous vs parous); number of pregnancies, number of childbirths	70(8) years	Cognitive performance, MRI, PiB PET	• Women with ≥ 5 children exhibited lower MMSE scores vs. < 5 children. • Women with ≥ 5 children had lower gray matter volume, especially in the hippocampus, vs. < 5 children. • No associations with amyloid plaque load or white matter hyperintensity volume.
Ning et al., 2020	Europe	303,196 male and female (160,077 females)	Yes	N/A	Number of children	57(8) years	Cognitive performance, MRI-derived brain age	• Men and women with ≥ 1 children exhibited faster response time and better visual memory vs. no children. • Parous individuals had a younger relative brain age on MRI.
Song et al., 2020	China	9,656 women	Yes	Range 5–23 years	Number of children, age at first birth, oral contraceptive usage	78(7) years	Change in cognitive performance	• Women with ≥ 5 children had reduced cognitive performance vs. 1–2 children. • Short duration of oral contraceptive usage associated with decreased cognitive impairment. • No association with age at first birth.
Schelbaum et al., 2021	United States	99 cognitively normal women	Yes	N/A	Number of pregnancies and children, hormonal contraceptive use	52(6) years	Cognitive performance; gray matter volume (GMV) on MRI	Number of children and pregnancies, and oral contraceptive use were associated with larger GMV in regions vulnerable to AD.

Some studies report better cognitive performance in midlife (Henderson et al., 2003; Ning et al., 2020), and a lower AD risk in later life (Fox et al., 2018) in women who had experienced pregnancy. Two large European studies reported less apparent brain aging in parous compared to nulliparous women, especially in association with a higher number of childbirths, as predicted using MRI-based machine learning models (de Lange et al., 2020; Ning et al., 2020). Studies of midlife women have also reported positive effects of parity on cognitive performance, such as improved verbal and visual memory performance (Henderson et al., 2003; Ning et al., 2020), and on GM volume in frontal and temporal regions (Schelbaum et al., 2021). While number of children was not directly associated with cognitive performance, GM volume in temporal cortex positively correlated with memory and global cognition, suggesting possible mediation effects (Schelbaum et al., 2021). Additionally, in studies that examined gravidity (total number of pregnancies including stillbirth, miscarriage, and/or abortion), elderly women who spent more cumulative months pregnant and breastfeeding over the course of a lifetime had a reduced risk of AD (Fox et al., 2013, 2018). Longer duration of breastfeeding was also found to be protective against AD dementia in other studies (Heys et al., 2011). As estrogen levels are lower during lactation, other factors are likely involved.

However, while some studies indicate that having 1–3 children may provide protective effects against dementia (Heys et al., 2011; Ning et al., 2020; Song et al., 2020), grand multiparity (5 or more pregnancies) may have detrimental effects instead (Rasgon et al., 2005a; Bae et al., 2020; Song et al., 2020; Figure 2E).

Findings of no associations between number of children and memory performance or AD and dementia risk were also reported (Ptok et al., 2002; Corbo et al., 2007; Ryan et al., 2009; Bae et al., 2020). The largest epidemiological study to date, the Rancho Bernardo Study in southern California, studied 1,025 women between the ages of 44–99 followed for over 28 years, with cognitive testing data collected at up to 7 different timepoints (Ilango et al., 2019). Results indicated no long-term influence of pregnancy history on age-related cognitive function, except for a slight decline in verbal memory in parous vs. nulliparous women, which did not survive correction for multiple comparisons (Ilango et al., 2019).

On the other hand, in some studies, parous women had greater cognitive decline on Mini-Mental State Examination (MMSE) scores (McLay et al., 2003), higher AD risk (Colucci et al., 2006) and a younger age at AD onset as compared to nulliparous women (Ptok et al., 2002), effects that seemed, however, limited to APOE-4 non-carriers (Corbo et al., 2007). A post-mortem study reported greater AD-related neuropathology with more childbirths, though no clear associations were observed with cognitive function (Beeri et al., 2009).

Discrepancies among studies may be due to small sample sizes (Corbo et al., 2007; Beeri et al., 2009), differences in cognitive assessments (Ryan et al., 2009) or diagnostic criteria (Ptok et al., 2002; Beeri et al., 2009; Bae et al., 2020), possible inclusion of non-biological children (Bae et al., 2020), and different exposure variables including parity (parous vs. nulliparous) (Ptok et al., 2002), gravidity (Colucci et al., 2006; Fox et al., 2018), number of children (Rasgon et al., 2005a; Heys et al., 2011), number of living childbirths (Bae et al., 2020; de Lange et al., 2020; Ning et al., 2020), or number of months spent pregnant (Fox et al., 2018). Associations with cognition were more commonly observed when parity was defined as number of childbirths or time spent pregnant rather than as having vs. not having children. Additionally, few studies have taken other pregnancy-related factors, such as age at first childbirth, breastfeeding, pregnancy complications such as pre-eclampsia and gestational diabetes, and oral contraceptive usage into consideration. Contrasting results might also be due to the timing of the observations, as the effects of motherhood on the brain are likely more discernible closer in time to childbirth than in older age or at post-mortem. Generally, studies that examined associations between number of children and cognitive performance closer to the time of childbirth have more consistently reported beneficial effects of pregnancy on brain and cognition function (Brunton and Russell, 2008).

## Exogenous Estrogen Exposures

### Hormonal Therapy for Menopause

If estrogen depletion in midlife is associated with increased risk of AD in later life, then theoretically estrogen therapy could reduce risk of AD. Although an increasing number of studies have explored menopausal HT for AD risk reduction, data regarding this issue is mixed. There are strong discrepancies between basic science, observational studies, and small clinical trials of HT on the one hand, and large randomized clinical trials of HT on the other hand.

The former studies generally reported a protective effect of HT on cognitive function and AD risk (Kawas et al., 1997; Zandi et al., 2002; Paganini-Hill et al., 2006; Sherwin, 2006; Whitmer et al., 2011), especially among younger, 50–59 year-old women (LeBlanc et al., 2001). Positive effects were more consistent with estrogen-only, or unopposed HT, for hysterectomized women (Sherwin and Phillips, 1990; Henderson et al., 2005; Rocca et al., 2007, 2010; Whitmer et al., 2011; Shao et al., 2012). These results are in contrast with large randomized, placebo-controlled clinical trials of HT, starting with the Women’s Health Initiative (WHI), a 15-year study tracking over 161,800 healthy, postmenopausal women. The study included two trials, the WHI Estrogen-plus-Progestin Study, in which women with a uterus were randomly assigned to receive either HT containing both estrogen and a progestin (Prempro) or a placebo; and the WHI Estrogen-Alone Study, in which women without a uterus were randomly assigned to receive either HT containing estrogen alone (Premarin) or a placebo. Cumulatively, the WHI has shown some benefits related to use of HT, including one-third fewer hip and vertebral fractures, and one-third lower risk of colorectal cancer relative to placebo (Rossouw et al., 2002; LaCroix et al., 2011). However, both trials were interrupted early as it was determined that both types of therapy were associated with specific health risks, particularly an increased risk of coronary artery disease, stroke and blood clots (Rossouw et al., 2002; Anderson et al., 2004). In both trials, risk of heart disease returned to normal levels after treatment discontinuation (Shumaker et al., 2003; Heiss et al., 2008). Additionally, the Estrogen-plus-Progestin arm of the study showed an increased risk of cancer (Rossouw et al., 2002; Anderson et al., 2004).

The WHI included an additional arm, the WHI Memory Study (WHIMS), which examined HT for dementia prevention in postmenopausal women ages 65 or older (Shumaker et al., 2003). Instead, results indicated a doubling of the risk of all-cause dementia among women in the Estrogen-plus-Progestin arm (Shumaker et al., 2003), and no significant effects in the Estrogen-Alone arm (Espeland et al., 2004; Shumaker et al., 2004). A major problem with this study is that participants were several years past menopause at enrollment, thus possibly already harboring pre-existing dementia or cardiovascular conditions. Re-examination of the WHI data indicates that the efficacy of HT is linked to the timing of initiation with respect to age at menopause onset (LeBlanc et al., 2001; Manson et al., 2006; Maki, 2013; Bove et al., 2014). On meta-analysis, younger, 50–59 year-old women who used HT had a 30–44% reduction in AD risk as compared to never-users (LeBlanc et al., 2001; Maki, 2013).

In the Early versus Late Intervention Trial with Estradiol (ELITE), HT reduced the progression of subclinical atherosclerosis when therapy was initiated soon after menopause (Hodis et al., 2016), which has been linked to a 30% reduced number of heart attacks and cardiac deaths (Salpeter et al., 2009). The newer ELITE-cog and Kronos Early Estrogen Prevention Study (KEEPS) trials have so far reported no adverse effects of HT on cognition among recently postmenopausal women, though no beneficial effects were observed either (Gleason et al., 2015; Henderson et al., 2016; Miller et al., 2019).

Clinical trials using brain scans as a secondary endpoint lend support to the hypothesis that both age at treatment and type of HT are important factors to consider. Some studies indicated positive effects of HT on GM volume (Erickson et al., 2005; Boccardi et al., 2006), CBF and CMRglc in AD-vulnerable regions, which have been attributed to effects of estrogen on the cerebrovascular system (Eberling et al., 2000; Maki and Resnick, 2000; Słopień et al., 2003; Rasgon et al., 2005b,^2014^; Silverman et al., 2011), with unopposed HT being more beneficial than combined HT (Silverman et al., 2011; Rasgon et al., 2014). Further, oral conjugated equine estrogen (CEE) therapy was associated with reduced GM volume (Resnick et al., 2009; Zhang et al., 2016), increased ventricular enlargement and WM hyperintensity load as compared to transdermal estradiol (Kantarci et al., 2018).

### Hormonal Contraceptives

Despite the millions of women worldwide using HC, little is known about HC effects on the brain (Petitti, 2003; Christin-Maitre, 2013; Taylor et al., 2019). Systemic HC have been available since the 1960s, sold under various brand names and formulations, most of which are administered orally, transdermally, transvaginally or via implants. HC are comprised of synthetic sex hormones, often ethinylestradiol, a more potent form of endogenous estradiol and synthetic progestin, which can either be androgenic or anti-androgenic (Pletzer et al., 2019; Taylor et al., 2021). The functions of HC treatment involve several mechanisms that lead to the inhibition of follicular development, suppression of the production of endogenous estradiol and progesterone, and preventing ovulation (Taylor et al., 2021).

Although studies investigating the impact of HC on later life cognition are scarce, 56% of the studies report a reduced risk of cognitive impairment (Li et al., 2016; Song et al., 2020) or higher scores on cognitive tests in midlife women taking HC (Egan and Gleason, 2012; Karim et al., 2016; Figure 2F). One study reported an almost 50% reduced risk of cognitive impairment in elderly women aged 60 or older who had used HC compared to never users (Li et al., 2016). Structural MRI studies of young adult women generally show greater GM volume in HC-users compared to natural cycling women in the hippocampus, parahippocampal and fusiform gyri, and cerebellum (Pletzer et al., 2010, 2015, 2019; De Bondt et al., 2013), although results are not always consistent (Petersen et al., 2015; Lisofsky et al., 2016). Among midlife women at risk for AD, HC users exhibited greater GM volume in medial temporal lobe, precuneus, fusiform gyrus, parietal and frontal cortex as compared to never users (Schelbaum et al., 2021).

The remaining studies reported no associations between HC use and dementia incidence (Najar et al., 2020), cognitive decline (McLay et al., 2003), or cognitive performance (Ryan et al., 2009; Tierney et al., 2013). Inconsistent findings may be a result of several factors such as age of initiation, HC formulations, dosage and duration of use (Taylor et al., 2021).

### Endocrine Therapy for Breast Cancer

Every year, 1.4 million women worldwide are diagnosed with breast cancer, which still results in over 400,000 deaths annually (Kamangar et al., 2006). Nearly 13% of American women are diagnosed with breast cancer at some point in their life (Branigan et al., 2020). While breast cancer is a multi-factorial disease, it has a known hormonal component. Around 75% of all breast cancers are hormone-receptor positive (HRP) and patients are usually advised to undergo adjuvant endocrine therapy. In this section, we review data on the effects of anti-estrogen therapy on cognition, as treatment with estrogen blockers and aromatase inhibitors (AIs) suppresses estrogenic function in body and brain (Zwart et al., 2015). This has spurred concerns around dementia risk in breast cancer survivors.

Two of the most frequently prescribed classes of endocrine therapy for breast cancer are selective estrogen receptor modulators (SERMs) (e.g., tamoxifen and raloxifene), and steroidal (i.e., exemestane) and non-steroidal AIs (i.e., anastrozole and letrozole). SERMs have tissue-specific agonistic and antagonistic actions on estrogen receptors, while AIs inhibit estrogen production in body and brain. Premenopausal women with HRP cancer typically receive tamoxifen whereas postmenopausal women receive AI monotherapy, or AI as adjuvant or neoadjuvant therapy with tamoxifen (Arnedos et al., 2015). Both therapies can include adjuvant chemotherapy depending on clinicopathological indications (Arnedos et al., 2015).

Owing to the increasing awareness of the importance of estrogens for brain health, more research is now focused on evaluating the potential adverse impact of endocrine therapies on cognition and AD risk in patients with breast cancer (Zwart et al., 2015). Several studies have indicated that chemotherapy with or without endocrine therapy can induce cognitive changes, especially declines in memory, processing speed, and executive function, as compared to pre-treatment levels (Wefel et al., 2015). Cognitive declines have been reported in 20–60% of chemotherapy-treated cancer survivors (Wefel et al., 2015), who also tend to exhibit GM loss and alterations in structural connectivity (de Ruiter et al., 2012). However, as many patients in these studies received both chemotherapy and endocrine therapy, the specific influence of hormonal treatment on cognition remains unclear. Herein, we review the studies that specifically addressed the cognitive effects of endocrine therapy in patients with breast cancer.

To date, three large intervention trials have conducted sub-studies to examine the effects of endocrine therapy on cognitive function in women with breast cancer. In the Arimidex Tamoxifen Alone or in Combination (ATAC) trial, five years of therapy with anastrozole alone, tamoxifen alone, and the combination of anastrozole and tamoxifen was associated with significantly lower scores on verbal memory and processing speed assessments in postmenopausal women with invasive operable breast cancer who had completed primary therapy and eligible for adjuvant hormonal therapy (including 20–22% on chemotherapy) relative to an age-controlled group of cancer-free women (Baum et al., 2002). A second analysis of the ATAC study compared cancer patients who had received surgery and various endocrine treatments with controls. None of the patients had done chemotherapy, and 67% were treated with course of radiotherapy. Results showed that patients receiving anastrozole, tamoxifen and combined treatment performed significantly worse on tasks of verbal memory and processing speed as compared to an age-controlled group of cancer-free women, independent of prior HT use (Shilling et al., 2003). No information was provided as to whether these effects differed by type of endocrine therapy.

The Tamoxifen Exemestane Adjuvant Multinational (TEAM) study examined breast cancer patients who commenced treatment within 10 weeks of completion of surgery and chemotherapy if indicated (nearly 2/3 for both tamoxifen followed by exemestane and exemestane alone groups did not have adjuvant chemotherapy) and cancer-free controls (van de Velde et al., 2011). Direct comparison of the use of tamoxifen or exemestane revealed a significant decline in verbal memory and executive functioning, especially processing speed, with tamoxifen use, and no negative effects of exemestane relative to controls (Schilder et al., 2010). These outcomes were more pronounced in patients aged 65 and older, suggesting a possible age-dependent effect of tamoxifen on cognition. Follow-up one year after cessation of therapy demonstrated improvement in cognitive scores for all groups.

Finally, the Breast International Group 198 study (BIG 198) (Thürlimann et al., 2005) examined postmenopausal patients with breast cancer who had been randomized to receive tamoxifen, letrozole, tamoxifen followed by letrozole, or letrozole followed by tamoxifen (Phillips et al., 2010). Some of these patients also received chemotherapy (24% for tamoxifen group, 35% for letrozole group). Using a cognitive composite score, results indicated significantly higher cognitive performance in the letrozole arm compared with the tamoxifen arm, although both groups performed below age-adjusted norms on the majority of tests (Phillips et al., 2010). Additionally, a neuroimaging study of postmenopausal women found that older women currently taking tamoxifen had smaller hippocampal volumes as compared to women taking unopposed estrogen therapy for menopause (Eberling et al., 2004).

Further investigation to account for menopause HT use, menopausal status, age since menopause, and pre-treatment hormonal status is warranted. Nonetheless, clinical trial results are consistent with the few observational studies that examined the influence of tamoxifen on cognition in truly chemotherapy-naive patients. Although limited due to small sample sizes, unmatched ages, and mixed treatment durations, these studies consistently showed negative effects of tamoxifen on verbal memory and fluency (Collins et al., 2009; Lejbak et al., 2010; Boele et al., 2015). In summary, tamoxifen, but not AIs, may be associated with negative cognitive effects in postmenopausal women. However, these effects appear to be temporary as improvements in cognitive function after therapy completion have been described (Paganini-Hill and Clark, 2000; Schilder et al., 2010).

Only two studies have specifically examined endocrine therapy independent of chemotherapy or radiation therapy as a risk factor for AD. One study reported a decrease in AD incidence in treated vs. untreated breast cancer patients (Branigan et al., 2020), while another study showed no difference in dementia incidence (Ording et al., 2013).

Other studies to date focused on mixed treatment populations, and reported conflicting findings of no associations with dementia (Ording et al., 2013; Bromley et al., 2019; Thompson et al., 2021), an increased risk of dementia (Liao et al., 2017), or a reduced risk of mild cognitive impairment (Yaffe et al., 2005), AD (Breuer and Anderson, 2000; Branigan et al., 2020) and dementia (Sun et al., 2016; Blanchette et al., 2020) with SERM and/or AI use. In two studies, SERM-treated breast cancer patients over the age of 70 had 12–18% lower risk of AD or dementia compared to untreated patients (Sun et al., 2016; Branigan et al., 2020). The protection associated with the SERMs was exclusively due to tamoxifen, and not to raloxifene (Branigan et al., 2020). Additionally, older patients receiving the steroidal aromatase inhibitor exemestane had a statistically significant decrease in the incidence of AD and dementia compared with patients receiving the non-steroidal therapies anastrozole and letrozole (Branigan et al., 2020).

Discrepancies among studies may be due to different assessments of outcome (Paganini-Hill and Clark, 2000; Sun et al., 2016), small sample sizes (Shilling et al., 2003; Palmer et al., 2008), differences in subject age (Palmer et al., 2008; Branigan et al., 2020), differences in treatment dose, duration, or type (Yaffe et al., 2005), or other confounding variables such as surgeries or chemotherapy (Eberling et al., 2004; Branigan et al., 2020). Future studies are needed to better characterize the relationship between endocrine therapy and AD risk in women.

### Gender-Affirming Hormone Therapy

Transgender people experience gender dysphoria due to incongruence between their gender identity and the sex they were assigned at birth (Winter et al., 2016). Gender-affirming hormone therapy (GHT) is the primary intervention sought by transgender people. GHT allows the acquisition of secondary sex characteristics more aligned with an individual’s gender identity, in part by reducing characteristics of biological sex (Winter et al., 2016). The mainstay of this lifelong treatment in transgender men is testosterone, typically delivered as intramuscular testosterone undecanoate or ester formulation (Irwig, 2017). Transgender women typically receive oral or transdermal estrogen preparations often in conjunction with a gonadotropin-releasing hormone analog or an anti-androgen (Tangpricha and den Heijer, 2017).

Evidence for negative effects of same-sex hormone deprivation on cognitive aging and AD risk begs the question of whether GHT impacts cognitive functioning in transgender individuals. While neuropsychological research on this topic is limited, meta-analysis indicates no adverse effects of GHT in young adult transgender men or women (Karalexi et al., 2020). Rather, young transgender men exhibited a significant enhancement of visuospatial ability following testosterone treatment (Karalexi et al., 2020).

GHT effects on cognition in older individuals are less clear as only one study of transgender women has investigated the long-term effects of GHT (van Heesewijk et al., 2021). This study compared 37 transgender women receiving GHT for at least 10 years (range 10–42 years) to an age and education level matched cohort of 222 cisgender women and men, showing that transgender women performed similar to cisgender men on all tests, but scored lower than cisgender women on immediate and delayed recall tests (van Heesewijk et al., 2021). No long-term studies of transgender men have been published.

Brain imaging studies thus far are also limited to transgender young adults (Guillamon et al., 2016). MRI studies of transgender women demonstrate that estradiol plus antiandrogen therapy was associated with a decrease in brain volume and an increase in ventricular volume after four to six months of treatment (Pol et al., 2006; Zubiaurre-Elorza et al., 2014), which persisted after at least one year (Spizzirri et al., 2018). Cortical thickness was also reduced post-treatment, with the strongest changes observed in occipital and parieto-temporal regions (Zubiaurre-Elorza et al., 2014). On the contrary, four months of androgen treatment increased total brain volume, total gray matter volume, and hypothalamic and thalamic volumes, as well as cortical thickness in parieto-occipital regions of transgender men (Pol et al., 2006; Zubiaurre-Elorza et al., 2014), which correlated with the increase in serum testosterone levels (Zubiaurre-Elorza et al., 2014). Additionally, 7 months of testosterone treatment increased fractional anisotropy in the superior longitudinal fasciculus and corticospinal tract of transgender men (Rametti et al., 2012). These increases might be due to anabolic effects of testosterone and its reduced metabolite dihydrotestosterone, which bind androgen receptors and to a lesser extent estrogen receptors (Negri-Cesi et al., 1996). The suppression of testosterone via antiandrogens in transgender women might therefore diminish the anabolic tone in brain tissues and induce decreases in volume (Zubiaurre-Elorza et al., 2014). Epidemiological data indicates that the use of estrogens in transgender women confers an increased risk of myocardial infarction and ischemic stroke, whereas transgender men receiving testosterone lack any consistent evidence of increased risk of cardiovascular or cerebrovascular disease (Connelly et al., 2019).

Overall, limited research indicates that GHT impacts the morphology as well as the connectivity of the brain in young transgender adults, possibly aligning their brains to the morphological characteristics of the desired gender. These changes do not appear to have negative effects on cognition in the short term. Short and long-term studies of older transgender people are lacking.

## Conclusion

Worldwide populations are aging and with advanced age is an increased risk for neurodegenerative diseases, including AD. While for many years the prevailing view held women’s greater longevity relative to men as the main reason for women’s higher AD prevalence (Seshadri et al., 1997; Hebert et al., 2001), there is increasing evidence that cerebral aging in women does not follow the same chronology as in men, and that female-specific reproductive history factors have a significant impact on later life cognition and AD risk.

There is growing consensus that, to stem the AD epidemic, sex-specific interventions that can potentially slow or reverse the trajectory of AD earlier in the course of the disease will be required. Recent evidence that AD starts in midlife (Sperling et al., 2013), and that the timing of the menopause transition coincides with the prodromal phase of AD, has highlighted a previously overlooked connection between menopause and AD risk. Currently, menopause is the most widely investigated female-specific risk factor for AD (Rahman et al., 2020). The neuroprotective effects exerted by estrogens have been proposed as a major reason for reduced signs of cerebrovascular and metabolic aging in premenopausal women compared to men (Raz, 2014; Zárate et al., 2017). When estrogen withdrawal occurs during menopause, effects of accelerated cellular aging become evident in the CNS and in the periphery, possibly leading to an increased risk of neurodegenerative events and AD (Wang et al., 2020a; Mosconi et al., 2021).

While very little work has been done to investigate changes in cognition and AD biomarkers during the transition to menopause, recent translational neuroimaging studies have provided *in vivo* evidence for emergence of AD endophenotypes in midlife women (Mosconi et al., 2017a,b, 2018, 2021; Rahman et al., 2020). Surgically induced menopause is associated with higher risk of AD and of AD biomarkers, especially in presence of an earlier age at oophorectomy (Bove et al., 2014). Menopause-related midlife deficits in glucose metabolism and activation of amyloidogenic processes could be early indicators of prodromal AD contributing to the sex difference in AD prevalence.

Altogether, clinical and biomarker data suggest an earlier start of AD pathogenesis in women than men, with onset in the perimenopause. The evidence for a connection between estrogen deprivation and AD risk has prompted investigation of additional indicators of estrogen exposure as predictors of dementia. Epidemiological studies to date have yielded conflicting results regarding estrogen exposure and dementia risk as proxies of lower lifetime estrogen exposure, such as later age at menarche and younger age at menopause, have been shown to be associated with increased, reduced, and null risk of cognitive impairment, dementia and AD in later life. While results on parity, pregnancy and number of children are mixed, recent neuroimaging data indicates a protective role of pregnancy against AD-biomarker risk in women, but not men (Schelbaum et al., 2021). This suggests that the biological process of pregnancy and the hormonal changes that accompany it, rather than lifestyle factors involved with raising children, may be associated with AD risk, though the exact relationship remains to be elucidated. More studies with larger and more diverse samples, neuroimaging biomarkers, and longer follow-up periods are needed to clarify the relationships between cumulative estrogen exposure and AD risk in women.

Among exogenous estrogen exposures, menopausal HT use has been heavily scrutinized due to the disparity between basic science, observational studies, and large randomized clinical trials. HT action on brain is dependent on multiple factors, including chronological age, stage of reproductive aging, duration of hypogonadism, and presence of symptoms, as well as the formulation of HT, route of administration, and the health status of the brain. There is mounting evidence that HT use in midlife may help sustain neurological health and reduce the risk of AD (Brinton, 2008), whereas HT initiated >5 years after menopause may be less beneficial if not detrimental as clinical studies with elderly women have shown (Shumaker et al., 2003). While more work is needed, recent neuroimaging data in midlife women also suggests that HT use near the time of menopause may have positive effects on brain aging (Mosconi et al., 2017a,b, 2018, 2021; Rahman et al., 2020; Schelbaum et al., 2021). Currently, menopause HT is not recommended for AD prevention or cognition preservation in women of menopausal age. Personalized physician advice which takes into consideration key factors including age, menopausal stage, symptoms, and comorbidities, may offer a greater look at how HT impacts AD risk as compared to the one-size-fits-all approach of randomized clinical trials, and argues for a precision medicine approach to HT use (Kim and Brinton, 2021; Kim et al., 2021).

While studies of HC use in midlife and elderly women are scarce, there is evidence for positive associations between history of HC use and cognitive function (Egan and Gleason, 2012; Li et al., 2016). In structural MRI studies, women at risk for AD who had ever taken HC exhibited larger regional GM volumes as compared to never users (Schelbaum et al., 2021), which may be attributed to estrogen receptor mediated increase in synaptic spine density (Pletzer and Kerschbaum, 2014). Given that over 100 million women are using HC, with the number increasing yearly (Taylor et al., 2021), studies investigating the long-term brain changes that accompany its use are vital for women’s health.

Neuropsychological studies and clinical trials of endocrine therapies for estrogen-positive cancer, including SERMs and aromatase inhibitors, suggest that adverse effects on cognition can exist, with and without chemotherapy, but may depend on the specific therapy used (Schilder et al., 2010). While not conclusive, a growing body of evidence from observational studies point to potential adverse effects of tamoxifen on cognition, whereas negative effects of aromatase inhibitor treatment are currently not consistent (van de Velde et al., 2011), though risks and benefits should be weighed for every patient. While studies linking anti-estrogen therapies to AD risk are scarce, the evidence so far indicates no clear increase in AD risk with tamoxifen and steroidal aromatase inhibitors, as one study found a decrease in AD incidence with endocrine therapy use (Branigan et al., 2020), while another found no association with dementia incidence (Ording et al., 2013). More work is needed to examine the effects of endocrine therapy on AD and dementia risk separately from those of chemotherapy, and to account for background hormonal milieu, previous HT and HC use, and age since menopause as important effect modifiers. Investigating the potential effects of anti-estrogen endocrine therapy for cancer on cognition is of increasing importance, as current guidelines permit the choice between different regimens.

While short-term studies reported no negative impact of GHT on cognition, evidence of the long-term impact of GHT on cognition is lacking, especially with respect to AD risk in older age. Risks may become more apparent with aging, and with longer duration of hormone use. Older transgender individuals are an especially vulnerable group, as they have higher risk of experiencing stress and depressive symptoms than cisgender individuals (Fredriksen-Goldsen et al., 2014), report higher rates of subjective cognitive decline (Flatt et al., 2018), and higher rates of social isolation and chronic illness (Fredriksen-Goldsen et al., 2018), all of which are risk factors for AD in turn.

Overall, sex steroid hormones are a long overlooked but critical contributor to cognitive aging. While the neurobiological consequences of reproductive history and hormonal changes on brain aging and AD risk have only begun to be understood, converging evidence supports a role for cumulative estrogen exposure in reducing risk of AD and dementia later in life. This strongly argues for continued examination of sex hormones and reproductive history factors in AD prevention strategies for women. There is an urgent need for prospective epidemiological, clinical and biomarkers studies with data taken at several time-points starting at midlife that examine the associations between cumulative estrogen exposure and cognitive function in later life. Understanding the dynamic interplay between sex, chronological aging, endocrine aging, and additional risk factors is crucial to inform and justify primary prevention strategies targeting female-specific risk factors underlying the increased prevalence of AD in women, and for development of future personalized preventive care.

## Author Contributions

LM, SJ, and NM discussed the concepts and wrote the manuscript. ES, GJ, EJ, KC, HH, SP, KN, SL-Z, YH, RI, and RB reviewed the literature and provided critical revision of the manuscript for important intellectual content. All authors contributed to the article and approved the submitted version.

## Conflict of Interest

The authors declare that the research was conducted in the absence of any commercial or financial relationships that could be construed as a potential conflict of interest.

## Publisher’s Note

All claims expressed in this article are solely those of the authors and do not necessarily represent those of their affiliated organizations, or those of the publisher, the editors and the reviewers. Any product that may be evaluated in this article, or claim that may be made by its manufacturer, is not guaranteed or endorsed by the publisher.
